# Erratum to “Next Generation Sequencing: Potential and Application in Drug Discovery”

**DOI:** 10.1155/2014/621354

**Published:** 2014-11-19

**Authors:** Navneet Kumar Yadav, Pooja Shukla, Ankur Omer, Shruti Pareek, A. K. Srivastava, F. W. Bansode, R. K. Singh

**Affiliations:** ^1^Hematological Facility, Division of Toxicology, CSIR-Central Drug Research Institute, BS-10/1, Sector 10, Jankipuram Extension, Sitapur Road, P.O. Box 173, Lucknow 226031, India; ^2^Academy of Scientific and Innovative Research, New Delhi 110 001, India; ^3^Jaipur National University, Jaipur, Rajasthan 302017, India; ^4^Computer Centre, CSIR-Central Drug Research Institute, BS-10/1, Sector 10, Jankipuram Extension, Sitapur Road, P.O. Box 173, Lucknow 226031, India; ^5^Division of Endocrinology, CSIR-Central Drug Research Institute, BS-10/1, Sector 10, Jankipuram Extension, Sitapur Road, P.O. Box 173, Lucknow 226031, India

Through this erratum the authors declare that two coauthors were missing in the article entitled “*Next generation sequencing: potential and application in drug discovery*”. The correct list of the authors and their affiliations are as shown above.

